# Strengthening capability for malaria research in Africa.

**DOI:** 10.3201/eid0707.017709

**Published:** 2001

**Authors:** F Zicker

**Affiliations:** World Health Organization, Geneva, Switzerland. zickerf@who.ch


### Strengthening Capability for Malaria Research in Africa

**Fabio Zicker**
World Health Organization, Geneva, Switzerland

The Task Force on Malaria Research Capability Strengthening (RCS) in Africa, coordinated by the United Nations Development Programme, the World Bank, and the World Health Organization (WHO) Special Programme for Research and Training in Tropical Diseases (TDR), represents a collaborative funding strategy in which multiple agencies and governments promote capacity-building activities in the context of the Multilateral Initiative for Malaria in Africa (MIM). This task force was established to promote human resource development and strengthen research capacity in malaria-endemic countries.

A total of 112 proposals involving 42 countries (25 from Africa) and over 200 partner institutions/research groups have been reviewed since the program's inception in 1998. The proposals covered a wide range of aspects of malaria research, including the clinical and molecular basis of drug resistance, drug policy, immune responses to *Plasmodium* infection, evaluation of natural products for antimalarial activity, parasite diversity, home management, vector biology, and epidemiology of malaria. The task force has recommended for funding, 23 full proposals involving 24 African and 8 European countries and the USA, with annual budgets ranging from US $60,000 to US $250,000. Twenty Ph.D. and 17 M.Sc. training grants were also approved in connection with the funded projects. In addition, support was also recommended for a few proposals that would promote interactions between partners for improving protocols and collecting preliminary data.

### Task Force Objectives

The objective of the malaria RCS grants is to develop or strengthen, core African research groups' (engaged in basic or applied science) capacity for producing effective control tools for malaria and improving relevant health policy strategies. The task force believes there is an urgent need to attract scientists with new skills to foster genuine partnerships based on national and regional priorities, mutual and complementary scientific objectives, expertise, and shared responsibility. The partnerships will also provide opportunities to study specific aspects of malaria at multiple sites.

### Research Priorities

The task force encourages research projects or programs to help establish networks focusing on the following areas, including multidisciplinary cross-cutting innovative approaches.

Address for correspondence: Fabio Zicker, UNDP/World Bank/WHO Special Programme for Research and Training in Tropical Diseases, Av Appia 20, 1211 Geneva, Switzerland; fax: 4122-791-4854; e-mail: zickerf@who.ch

Antimalarial drug policy and chemotherapy—develop ment of strategies for rapid mapping of drug resistance; innovative approaches for preventing, retarding, and reversing drug resistance; definition of criteria for replacing first-line drugs.

Epidemiology—the use of new technologies to identify parasite diversity and its effect on immune responses; development of methods to measure the impact of interventions; and development of simple and rapid epidemiologic methods for mapping malaria morbidity and mortality.

Pathogenesis and immunology—studies on parasite-vector-host factors involved in severe disease and malaria in pregnancy, with the aim of developing and promoting improved control and management strategies and of evaluating potential vaccine candidates.

Entomology and vector studies—screening of natural local products for insecticidal and repellent properties, application of molecular tools for studies on vector biology, feeding behavior, vector capacity, insecticide resistance, and population genetics with the aim of identifying and developing effective strategies for vector control in settings of low and high transmission.

Health systems research, including social science—improvement of the home management of malaria on the basis of community knowledge, community practices, and the development of new products.

Natural products and antimalarial drug development—promotion of systematic identification and chemical and biological screening with in vitro and in vivo systems for isolating antimalarial compounds from natural products used by the indigenous populations for treatment of fevers.

### Project Profile

Proposals should be submitted and coordinated by an African national scientist working in a research group in Africa, and should include at least two African research partner institutions (one established and one emerging) and at least one non-African partner, which could be an international institution with a base in Africa. All proposals must describe in detail a plan for strengthening research capability. The grants are awarded on the basis of scientific merit, relevance, and quality of partnerships that promote capacity building and human resource development in Africa.

### Enhancing Factors

Activities of the task force have been enhanced by the use of unique strategies for identifying and managing the research projects. These include the following:

Strengthening research capability through sound research projects.

Developing a select group of projects around the interface of bench work/control operations.

Focusing the research agenda in priority areas.

Maximizing training opportunities.

Working for more rapid funding and implementation.

Encouraging research collaboration among developing country scientists and among developing and developed country scientists.

Promoting higher funding levels, including salary supplementation, for principal investigators to enhance long-term career commitment to research in Africa.

### Task Force Progress

The activities of the MIM/TDR-RCS have produced the following results:

1. Continental networks for malaria mortality/demographic surveillance have been enhanced, including mapping the risk of malaria and determining the relationship(s) between transmission intensity and control activities in Africa. These networks have hubs in South Africa, Mozambique, Burkina Faso, and Ghana with 31 collaborating sites in 15 countries in Africa.

2. A model for collaboration between research scientists, malaria control personnel, and policy makers has been developed (Nigeria).

3. The impact of environmental modification for agricultural activities on malaria transmission and morbidity has been evaluated (Benin and Ivory Coast)

4. Simple molecular assays have been developed for the surveillance of drug-resistant malaria, and results have been used to make evidence-based malaria control policy decisions (Mali and Tanzania)

.

5. Information has been obtained on the contributions of tumor necrosis factor and soluble receptors to the pathogenesis of cerebral malaria and severe malarial anemia (Ghana).

6. National capacity has been strengthened in African countries for rational selection of insecticides used in vector control (South Africa, Benin.)

7. Biodiversity prospecting (discovery and development of biochemical and genetic resources from plants, animals, and microorganisms to be used in biotechnology applications) has begun for new antimalaria compounds and insecticides (Kenya and Nigeria).

8. Five African research centers have been established to develop strategies for rapid mapping and control of drug-resistant malaria (Ghana, Nigeria, Malawi, Mali, and Tanzania).

Other accomplishments of the MIM/TDR-RCS include conducting the following group learning activities between March 1988 and March 2000:

Workshop on Research Capacity Development in Africa, March 1999.

Workshop on Markers of Antimalarial Drug Resistance: Practical, Clinical, and Epidemiological Applications, June 1999.

Laboratory Training on Molecular Markers of Antimalar ial Drug Resistance, January-February 2000. Jointly organized by the MIM/TDR–RCS, National Institutes of Health (NIH), Malaria Research and Reference Reagent Resource Center (MR4), and Malaria Research Training Center, Bamako.

Workshop on Handling and Managing Biological Materials, March 2000. Jointly organized by the MR4, American Type Culture Collection, MIM/TDR-RCS, and Centre Nationale de Lutte Contre le Paludisme, Burkina Faso.

Mini-Symposium on Biological Resource Centers in Africa: Creating New Research Opportunities in Malaria, March 2000. Jointly organized by the MIM/TDR–RCS and MR4.

The success of this initiative will be confirmed by the existence of a sustainable malaria research community, which is capable of implementing relevant public health interventions and policies across Africa. The MIM/TDR program represents an innovative approach to accomplish this goal.

